# The Association Between Serum Lactate Concentration, Base Deficit, and Mortality in Polytrauma Patients as a Prognostic Factor: An Observational Study

**DOI:** 10.7759/cureus.28200

**Published:** 2022-08-20

**Authors:** Divya Jyoti, Anil Kumar, Talat Halim, Ahmed A Hai

**Affiliations:** 1 Emergency Medicine, Paras HMRI (Hai Medicare and Research Institute) Hospital, Patna, IND; 2 Trauma & Emergency, All India Institute of Medical Sciences Patna, Patna, IND; 3 Trauma Surgery, Paras HMRI (Hai Medicare and Research Institute) Hospital, Patna, IND; 4 Surgery, Paras HMRI (Hai Medicare and Research Institute) Hospital, Patna, IND

**Keywords:** injury severity score (iss), hypoperfusion, multiple organ dysfunction syndrome (mods), observational study, mortality, polytrauma, base deficit, serum lactate

## Abstract

Introduction

In polytrauma patients, it is crucial to identify the severity of the injuries to ensure patient safety and survival. Polytrauma leads to hypotension and hypoperfusion, which results in an anaerobic metabolism with acidosis and a decrease in base excess. Thus, blood lactate levels above a certain threshold indicate the existence of global tissue hypoxia, which is a precursor to shock and multiple organ dysfunction syndrome (MODS). The serum lactate and base deficit (BD) levels are used in polytrauma patients as measures of damage severity and resuscitation endpoints and as a way to evaluate therapy efficacy and to predict outcomes. Thus, arterial blood gas analysis is of great value in assessing the status and prognosis of patients with polytrauma. There are few comparative studies on the predictive values of these markers in trauma patients. To determine which measure can more accurately predict the prognosis of polytrauma patients, the present study investigated the predictive values of mortality of these indicators for mortality within 48 hours of admission to the emergency room (ER) in patients with polytrauma.

Methods

This prospective study was designed for a single tertiary care center in northern India. We included 90 patients with polytrauma who were between the ages of 18 and 70 years, with the exception of pregnant women, who presented to the ER within six hours of injury with an injury severity score (ISS) >16, serum lactate level >2.0 mmol/L, and BD -4.0 mEq/L at the time of admission. If the patient's ISS was >16 at the time of ER presentation, arterial blood samples were drawn to determine the serum lactate and BD level at the time of admission and at 12, 24, and 48 hours intervals after ER admission. The primary outcome was the change in serum lactate and BD level in polytrauma. The secondary outcomes were an association of serum lactate and BD with mortality and the correlation between serum lactate with the BD and ISS with mortality of polytrauma patients. The timing of all outcome assessments was at 48 hours after each patient's ER admission.

Results

Lactate clearance from 0-12 hours (t = 2.28, p <0.05), 0-24 hours (t = 6.01, p <0.001), and 0-48 hours (t = 7.98, p <0.001) and a correction in BD from 0-24 (t = 2.68, p <0.01 ) and 0-48 hours (t = 5.46, p <0.001) were significantly higher in nonsurvivors as compared with survivors. In survivors and nonsurvivors, mean serum lactate levels (2.46 ± 1.46 versus 4.15 ± 2.99, t = 3.31, p <0.001, 95%Cl) and mean BD (-3.17 ± 2.58 versus -6.5 ± 4.91, t = 3.86, p <0.001, 95%CI) had a statistically significant difference. The serum lactate and BD levels at time of ER admission (r L0, BD0 = -0.765, p <0.01) and 48 hours after ER admission (r L48, BD 48 = -0.652, p <0.001) were highly negatively correlated.

Conclusion

In polytrauma patients, serum lactate and BD are simple, quick, and independent biochemical predictors of 48-hour mortality, and this single arterial blood test would thereby improve decision-making for resuscitation effectiveness. Prolonged lactate and BD normalization time were associated with higher mortality. Serum lactate and BD are negatively correlated. A higher ISS at admission was associated with a higher incidence of mortality in polytrauma patients.

## Introduction

In seriously injured trauma patients at high risk of mortality, it is crucial to identify the severity of the injury as soon as possible to ensure patient safety and lower medical risks [1,2]. The assessments determine patient triage and treatment that further focuses on reducing time spent at the scene of the accident as much as possible, as well as ensuring prompt and adequate oxygenation and enhancing organ perfusion to improve survival. Thus, an in-depth study of the prognostic indicators in patients with polytrauma is warranted. The injury severity score (ISS) is the most often used trauma score to indicate the severity of injury and has a strong link with the combination of hypoperfusion and coagulopathy linked to a higher risk of death. Polytrauma is life-threatening when severity of the injury ≥ 16 on the ISS [3].

Polytrauma leads to a significant drop in blood flow, hypoperfusion, and thus a critical curtailment in the delivery of important nutrients to sustain the metabolic requirements of organs and tissues occurs and causes tissue hypoxia. The body adopts an anaerobic metabolism if tissue perfusion is insufficient. Lactate is released into the blood in this type of metabolism, resulting in acidosis and a decrease in base excess. Serum L clearance is controlled not only by macrocirculation, but also by microcirculation ( the latter, a network of arterioles, capillaries, and venules), as well as by mitochondrial function, and is representative of tissue perfusion. Thus blood lactate levels above a certain threshold indicate the existence of global tissue hypoxia, which is a precursor to shock and multiple organ dysfunction syndrome (MODS) [4]. Lactate measurement may not fully reflect the amount of the underlying metabolic acidosis, despite the fact that it indicates the severity of decreased perfusion. BD, on the other hand, can measure the severity of both anaerobic and aerobic acidosis and may be a better clinical indicator for determining the metabolic acid-base balance. BD, but not L is linked to the development of trauma coagulopathy [5]. Despite the advances in trauma care in the last two decades, arterial BD remains a useful prognostic marker in trauma patients [6].

The lactate and BD from an arterial blood gas (ABG) could reflect the internal environment of patients and trauma patients' ABGs often show significant changes [7]. Serum lactate and BD levels are used in trauma patients as measures of severity of tissue damage and resuscitation endpoints, and as a way to evaluate therapy efficacy and predict outcomes [8]. Thus, ABG analysis is of great value in evaluating the condition and prognosis in patients with multiple trauma [9]. When established risk factors are taken into account, lactate-guided treatment dramatically reduces hospital mortality in patients with hyperlactatemia on admission. There are few comparative studies on the predictive values of these markers in the trauma population, despite previous research showing that these indicators might assess the condition and predict the prognosis [10]. 

## Materials and methods

Study participants

The study was conducted in the department of emergency medicine, Paras Hai Medicare and Research Institute (HMRI) Hospitals, Patna, India, from September 2019 to June 2021. In this prospective study, we included 90 adult patients between the ages of 18 to 70 years, with a history of trauma who presented to the ER within six hours of injury and had an ISS >16, serum lactate level >2.0 mmol/l, and BD <-4 mEq/L at the time of admission. We excluded those patients who died within 15 minutes of arrival to the ER. We also excluded pregnant patients and patients with a history of diabetes mellitus, coronary artery disease, renal failure, liver dysfunction, alcoholism, malignancy (especially lymphoma), epilepsy, seizures, acute pancreatitis, and regular therapeutic interventions such as epinephrine, metformin or nucleoside analogues used in treating human immunodeficiency virus (HIV). 

Collection of data

 At the time of admission, ISS was calculated. Arterial blood samples were collected for serum lactate and BD levels at the time of admission and at 12, 24, and 48 hours after admission. 

Outcome measures

The primary outcome was the change in the serum lactate and BD levels in polytrauma patients at the time of admission and at intervals of 12, 24, and 48 hours after admission. The secondary outcomes were: 1) The association of the serum lactate with the mortality of polytrauma patients, 2) the association of the BD with polytrauma patient mortality, 3) the correlation between serum lactate and BD in polytrauma patients, and 4) the correlation between ISS and mortality of polytrauma patients. The timing of all outcome assessments was for 48 hours.

Data management and statistical analysis

All data were collected into a customized pro forma and then transferred to Microsoft Excel (Microsoft Corporation, Redmond, Washington, United States). Statistical analysis was done on SPSS Statistics for Windows, Version 17.0 (Released 2008; SPSS Inc., Chicago, United States). All continuous variables were expressed as the mean ± standard deviation (SD). They were compared using a t-test for an independent sample. A p-value of 0.05 was deemed statistically significant. Final data were presented in the form of tables and graphs. The correlation between serum lactate and BD was calculated using Pearson’s correlation formula.

Ethical considerations

Before the commencement of the study, the study protocol was submitted to and approved by the Institutional Ethical Committee for Biomedical and Health Research, HMRI Paras Hospital, Patna, Bihar, India (IEC-B & H/PHMRI/Faculty/138/2021). The study is in accordance with the Declaration of Helsinki and followed the guidelines laid out by The Indian Council of Medical Research. Prior to the enrollment in this study program, written voluntary informed consent was obtained from the patients/legal guardians. The patients underwent the standard treatment protocol followed by the institution. In addition, the institute was not burdened in any way as a result of the current study.

## Results

**Figure 1:**
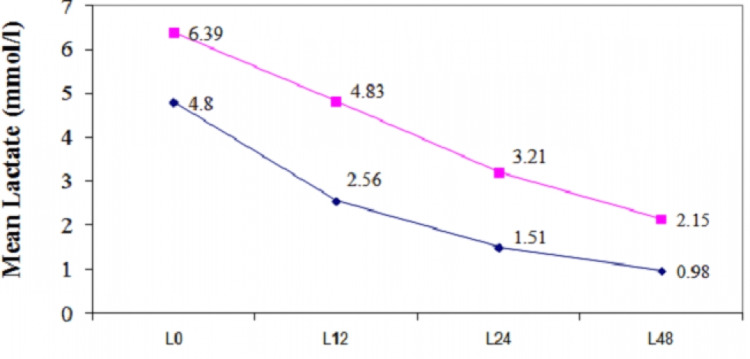
Serum lactate level trends in survivors (violet line) versus nonsurvivors (pink line) L0: lactate level at the time of ER admission; L12: lactate level at 12 hours after ER admission; L24: lactate level at 24 hours after ER admission, L48: lactate level at 48 hours after ER admission

The 90 polytrauma patients included in our study were >18 and <70 years of age and their ISS were >16. The patients' serial serum lactate and BD levels were recorded to assess their prognostic value. The majority of patients (41.1%) were in the age group of 20-29 years. Of the 90 polytrauma patients, 77 (86%) were male and 13 (14%) were female. Thus, males were predominant with no significant statistical difference between males and females in mean age in years (p-value >0.05). Road traffic accidents represented the major cause of polytrauma patients i.e., 78.9%. The mean age of survivors was 34.11 ± 10.57 years, while the mean age of nonsurvivors was 36.73 ± 15.91 years. The category of survivors had 67 males and eight females, while the nonsurvivors group had, 10 males and five females (t-test = 1.45, p-value < 0.05). The gender distribution of survivors and nonsurvivors was statistically significant (p <0.05). Mean serum lactate levels between survivors and nonsurvivors at admission (4.80 ± 3.03 versus 6.39 ± 3.62) Figure. 1) were not significantly different (p-value >0.05), but at 12 hours after ER admission (2.56 ± 2.63 versus 4.83 ± 5.22), 24 hours after admission (1.51 ± 1.05 versus 3.21 ± 2.75), and 48 hours after admission (0.98 ± 0.63 versus 2.15 ± 2.63) were statistically significantly different (p <0.01, p <0.001, and p <0.001, respectively) (Table 1). 

**Table 1:** Mean serum lactate levels versus outcomes at various times from ER arrival L0: lactate level at the time of ER admission; L12: lactate level at 12 hours after ER admission; L24: lactate level at 24 hours after ER admission, L48: lactate level at 48 hours after ER admission; mmol/L: millimoles per liter

Time of lactate level	Survivor lactate levels		Non-survivor lactate levels		T-Value	P-Value
	Mean (mmol/L)	SD (mmol/L)	Mean (mmol/L)	SD (mmol/L)		
L0	4.80	3.03	6.39	3.62	1.81	>0.05
L12	2.56	2.63	4.83	5.22	2.63	<0.01
L24	1.51	1.05	3.21	2.75	4.14	<0.001
L48	0.98	0.63	2.15	2.63	3.45	<0.001

**Figure 2:**
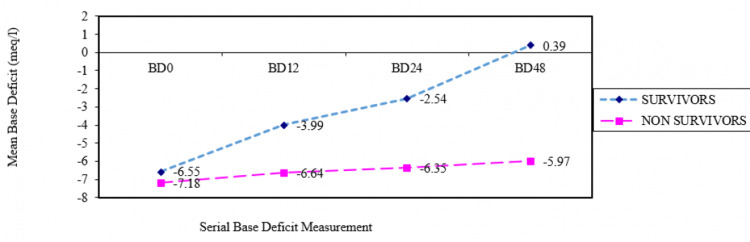
BD level trends in survivors (violet line) versus nonsurvivors (pink line) BD0: base deficit at time of ER presentation; BD12: base deficit at 12 hours after ER presentation; BD 24: base deficit at 24 hours after ER presentation; base deficit at 48 hours after ER presentation; BD: base deficit

The mean BD levels of survivors and nonsurvivors at the time of ER presentation (-6.55 ± 3.72 versus -7.18 ± 4.68) (Figure 2) and at 12 hours after ER presentation (-3.99 ± 4.54 versus -6.64 ± 6.99) were not statistically significantly different (p-value >0.05 and p >0.05, respectively) but mean BD levels of survivors and nonsurvivors at 24 hours after admission (-2.54 ± 3.79 versus -6.35 ± 7.12) and the values at 48 hours after admission (0.39 ± 2.53 versus -5.97 ± 4.99) were statistically significantly different (p <0.01 and p <0.001, respectively) (Table 2).

**Table 2:** Mean serum BD versus outcomes BD0: BD level at the time of ER admission; BD12: BD level at 12 hours after ER admission; BD24: BD level at 24 hours after ER admission; BD48: BD level at 48 hours after ER admission; BD: base deficit; mEq/L: milliequivalents per liter

BD at the time from ER arrival (hours)	Survivor BD levels		Non-Survivor BD level		T-Value	P-Value
	Mean (mEq/L)	SD (mEq/L)	Mean (mEq/L)	SD (mEq/L)		
BD0	-6.55	3.72	-7.18	4.68	0.57	>0.05
BD12	-3.99	4.54	-6.64	6.99	1.87	>0.05
BD24	-2.54	3.79	-6.35	7.12	3.14	<0.01
BD48	0.39	2.53	-5.97	4.99	7.36	<0.001

**Figure 3:**
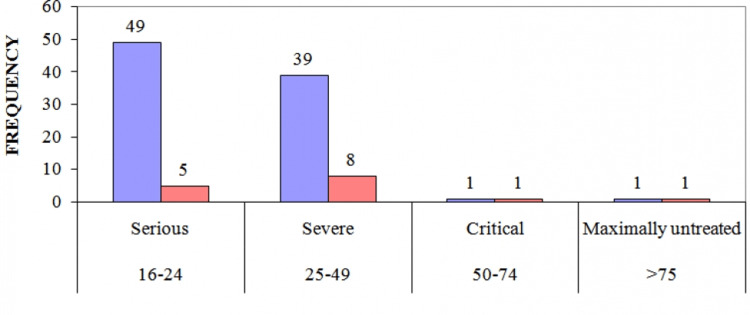
Severity score (violet bar) versus mortality (red bar)

Serum lactate levels among survivors and nonsurvivors at different times with respect to their point of ER admission were compared and lactate clearance from 0-12 hours (t = 2.28, p <0.05), 0-24 hours (t= 6.01, p <0.001), and 0-48 hours (t = 7.98, p <0.001) were statistically significantly higher in nonsurvivors when compared to survivors. Correction in BD from 0-12 hours (t = 1.60, p >0.05) were not statistically significantly higher but from 24 hours (t = 2.68, p <0.01) and from 0-48 hours (t = 5.64, p <0.001) were statistically significantly higher. The mean serum lactate and mean BD of survivor versus nonsurvivor were statistically significant (p <0.001) (Table 3). Using Pearson’s correlation formula, it was concluded that serum lactate and BD at the time of ER admission (r L0, BD0 = -0.765, p <0.01) and at 48 hours (r L48, BD48 = -0.652, p <0.001) were highly negatively correlated (i.e. both variables changes were in the opposite directions). There was a correlation between ISS and the mortality of trauma patients. A higher ISS at the time of ER admission was associated with the mortality of polytrauma patients (Figure 3).

**Table 3:** Mean and SD of the mean serum lactate and mean BD of survivors versus nonsurvivors BD: base deficit

	Survivors		Nonsurvivors		T-Value	P-Value
	Mean	SD	Mean	SD		
Lactate (mmol/L)	2.46	1.46	4.15	2.99	3.31	<0.001
BD (mEq/L)	-3.17	2.58	-6.5	4.91	3.86	<0.001

## Discussion

In our study of polytrauma patients, we found that early lactate normalization improves the outcome. The inability to lower lactate concentrations to normal raises the risk of mortality within the first 48 hours after ER admission. Serial lactate concentration measurement is a crucial indicator of the effectiveness of resuscitation efforts. In polytrauma patients, poor BDs were associated with adverse outcomes and a higher risk of death. The lactate and BD levels were highly negatively correlated with each other and are independent predictors of 48-hour mortality in polytrauma patients. A higher ISS at the time of admission was associated with mortality in polytrauma patients. The 90 polytrauma patients included in our study were >18 and <70 years of age and their ISS were >16. The patients' serial serum lactate and BD levels were recorded to assess their prognostic value.

In our study, the mortality rate was 17%, which is consistent with studies of seriously injured trauma patients, though the cited study evaluated only elderly patients [11]. Our study showed that both serum lactate levels at ER admission (p >0.05) and BD levels at admission (p >0.05) were not statistically significantly associated with mortality, which is consistent with the study done by Freitas et al. [12]. However, serum lactate level at 12 hours was significantly higher in nonsurvivors when compared to survivors (p <0.01), and the BD was found to be significantly higher in nonsurvivors when compared to survivors at 24 hours (P <0.01), which is consistent with the study done by Neville et al. [13].

We found that prolonged lactate normalization time was associated with higher mortality. The failure of a patient to normalize lactate is associated with a 100% mortality, which is consistent with the study done by Kunduri et al. [14]. Prolonged BD normalization time was associated with higher mortality in our study. This observation was in accordance with the study of Mutschler et al. published in 2013 [15]. In our study, serum lactate and BD were seen to be highly negatively correlated and that was statistically significant (p <0.001). This is in accordance with Davis et al. [16]. We found that serial lactate levels may perform better than serial BD in predicting mortality, which was confirmed by Martin et al. [17]. There was a correlation between ISS and the mortality of trauma patients in our study as found in the study conducted by Champion and colleagues [18].

Serum lactate and BD are prognostic markers of trauma patients as well as a guide to the resuscitation of polytrauma patients. Prolonged lactate and BD normalization time were associated with higher mortality. Thus an arterial BGA should be performed on every polytrauma patient. In polytrauma patients, serum lactate and BD levels are simple, quick, and independent biochemical predictors of 48-hour mortality, and this single test would thereby improve decision-making for resuscitation effectiveness. Serial lactate concentration measurements and early lactate normalization improved the outcome of polytrauma patients. The inability to lower lactate concentrations to normal raises the risk of mortality within the first 24 hours after ER admission. A poor BD was associated with adverse polytrauma patient outcomes and portended a higher risk of mortality. The lactate and BD levels are highly negatively correlated with each other and are independent predictors of 48-hour mortality for multiple trauma.

To assess the usefulness of serum lactate and BD for predicting mortality in polytrauma patients of all ages, including pregnant women and patients with co-morbidities, more research is required. The serum lactate and BD levels in patients with minor polytrauma should be studied. These two levels should be compared to or correlated with other acid-base pH variables, such as anion gap, strong ion difference, or strong ion gap, in further research. Our study was not comparable to the study done by Davis, et al., which concluded that BD levels are better than lactate levels for measuring resuscitation needs [16]. A study by Okello et al., concluded that even one raised value of lactate levels of ≥2.0 mmol/ significantly differentiated severely from non-severely injured patients that were not found by our study [19].

Limitations of the study

First, this study was conducted on an adult population and, hence, may not apply to pediatric patients. Second, our research was observational and, thus, we were able to show a connection but not prove causality. Third, because many of these patients were removed from our study due to a lack of lactate and/or BD values, our findings cannot be applied to minor trauma. Fourth, because other acid-base variables such as anion gap and strong ion gap are less commonly measured than blood lactate and BD, we did not evaluate them in our study.

## Conclusions

In polytrauma patients, serum lactate and BD are simple, quick, and independent biochemical predictors of 48-hour mortality, and this single test would thereby improve decision-making for resuscitation effectiveness. Prolonged lactate and BD normalization time were associated with higher mortality. A higher ISS at admission was associated with a higher incidence of mortality in polytrauma patients.
